# Supplementary material for: PFA ependymoma-associated protein EZHIP inhibits PRC2 activity through a H3 K27M-like mechanism
Source: Nat Commun. 2019 May 13;10:2146. doi: 10.1038/s41467-019-09981-6 (PMC6513997; doi:10.1038/s41467-019-09981-6)
Supplement: Supplementary file 1 — Supplementary Information [file 41467_2019_9981_MOESM1_ESM.pdf]

PFA ependymoma-associated protein EZHIP inhibits PRC2 activity through a H3 K27M-like mechanism

Jain *et al*.

Supplementary Information

Supplementary Figure 1

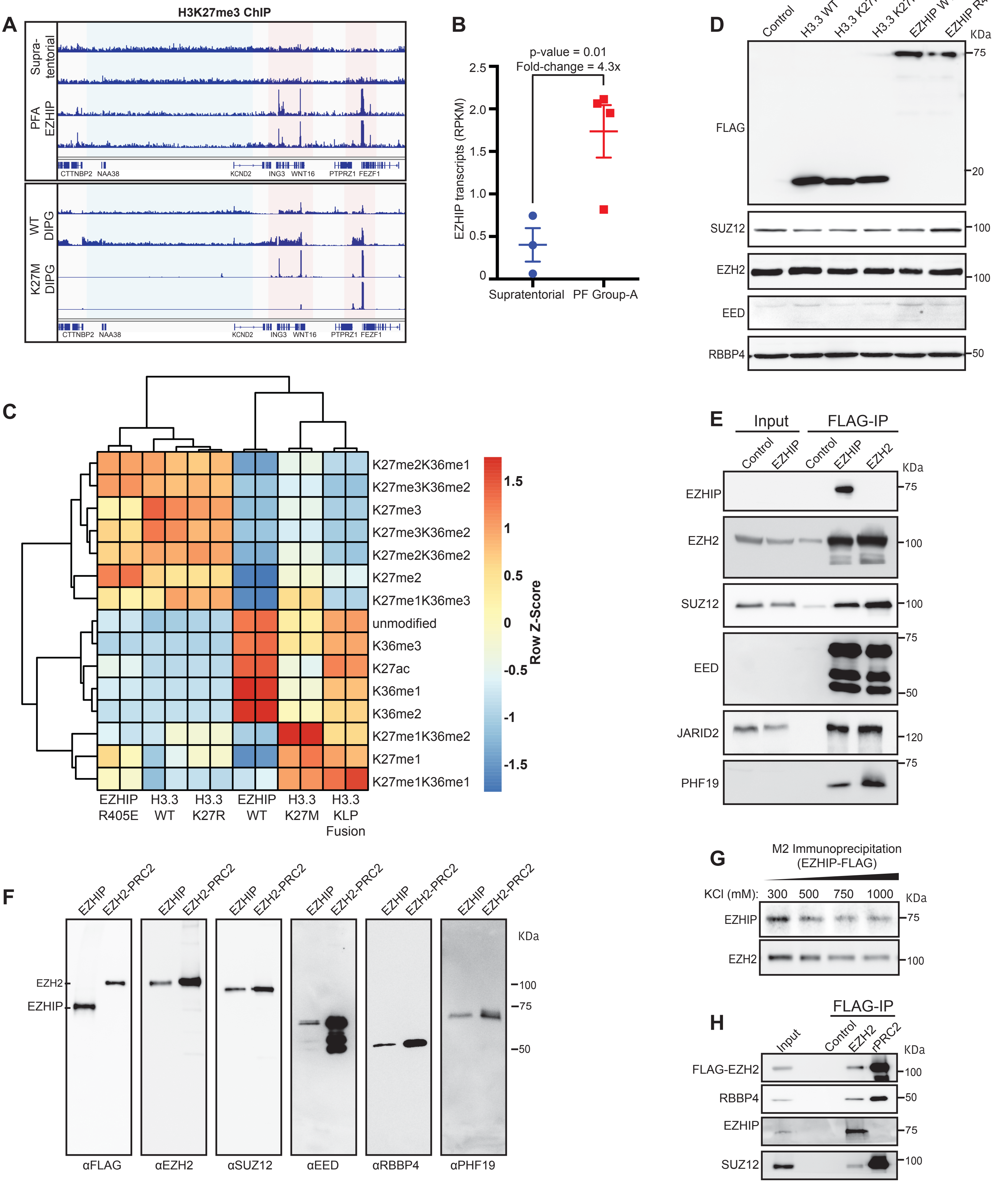

Supplementary Figure 1: EZHIP forms a stable complex with PRC2 and lowers H3K27me3 *in vivo*

(A) Genome viewer representation of ChIP-Seq for H3K27me3 in a 6 Mbp region of the human genome. Red shaded region represents residual H3K27me3; broad, intergenic H3K27me3 in blue. (B) Plot displaying the number of EZHIP transcripts found in PFA and supratentorial ependymomas. Reads were RPKM normalized. P-value was determined using non-parametric t-test. (C) Acid-extracted histones were subjected to bottom-up quantitative mass-spectrometry for measurement of histone PTM abundances. The abundances of modifications on H3K27 and H3K36 relative to the total peptide (as determined by the sum of all modifications) are plotted as heatmap. The relative abundances were z-score transformed. (D) Immunoblots from whole cells lysates of 293T cells expressing HA-FLAG-tagged H3.3 WT or K27M/R or FLAG-tagged EZHIP WT or R405E mutants. (E) Immunoblots of eluates from FLAG M2 immunoprecipitation from nuclear extracts of 293T cells expressing FLAG-tagged EZHIP or FLAG-tagged Ezh2. Nuclear extract from parental 293T cells was used as control. (F) M2 Immunoprecipitated material (EZHIP-FLAG or EZH2-FLAG) was subjected to anion-exchange chromatography. Immunoblots display the components of PRC2 in eluate corresponding to 350 mM KCl from anion exchange chromatography. (G) Immunoblots of eluates from FLAG M2 immunoprecipitation as in E, except with washes with buffers containing increasing concentrations of KCl. (H) Immunoblots of eluates from FLAG M2 immunoprecipitation from nuclear extracts of U2OS cells expressing FLAG-tagged EZH2. Nuclear extract was first incubated with unconjugated agarose beads, which was used as control.

Supplementary Figure 2

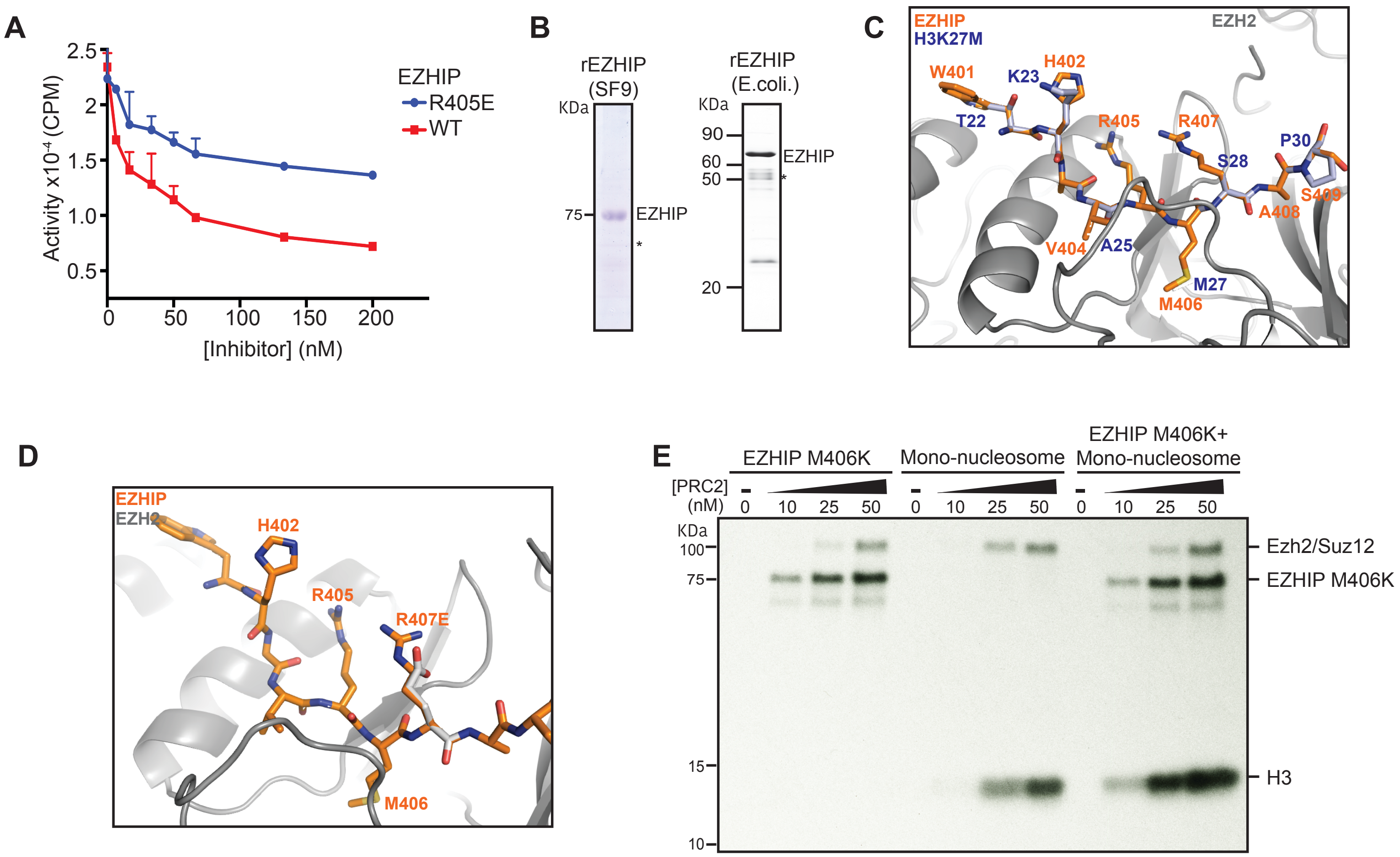

Supplementary Figure 2: EZHIP conserved peptide mimics H3K27M peptide

(A) Quantification of in vitro methyltransferase reactions with PRC2 and oligonucleosome substrate. Full length recombinant EZHIP WT or R405E mutant purified from E. coli was titrated into the reaction mixture as shown. Half of the reaction was subjected to SDS-PAGE followed by fluorography (Figure 2B) and the other half was used for quantification by scintillation counting. Error bars represent standard deviation. (B) Coommassie stained SDS-PAGE gel showing recombinant EZHIP purified from SF9 cells and BL21 E.coli. (C) Side-chains of H3K27M peptide in the PRC2-H3K27M co-crystal structure (pdb: 5HYN) were mutated to model side chains of EZHIP conserved peptide. (D) Position of EZHIP R407 away from EZH2 surface, towards the solvent in the modelled co-crystal structure. (E) 0.3 μM EZHIP M406K mutant or recombinant nucleosomes or both were incubated with increasing concentrations of recombinant PRC2 with 1 μM 3H-SAM and 25 μM H3K27me3 peptide for 1 hr. Reaction was subjected to SDS-PAGE followed by fluorography.

Supplementary Figure 3

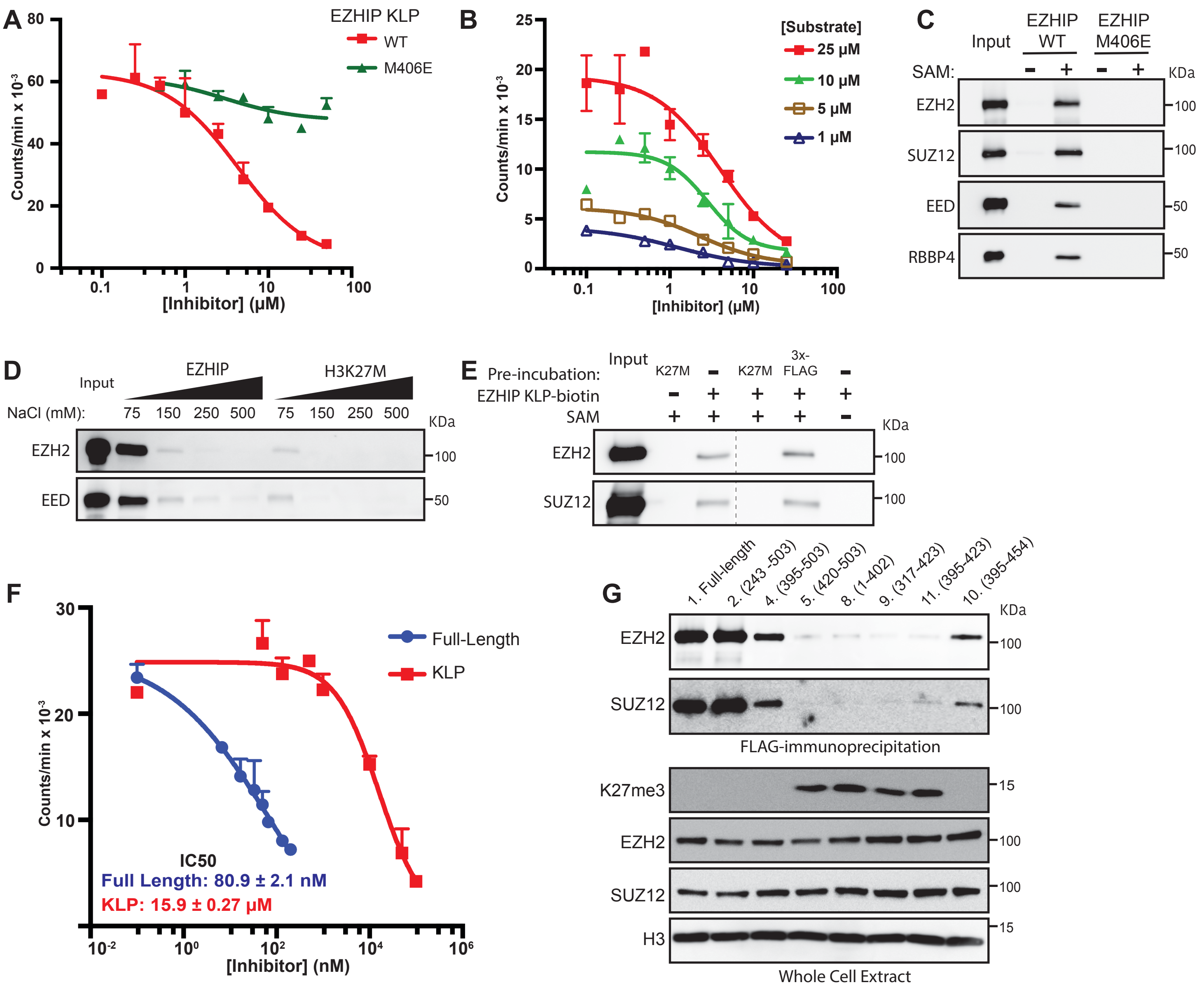

Supplementary Figure 3: "K27M-like" EZHIP peptide binds and inhibits PRC2

(A) In vitro PRC2 reactions using varying concentrations of H3 18-37 substrate peptides at increasing concentrations of K27M-like EZHIP 403-423 peptide. Variable slope, four parameter Hill curve was fitted to the data to determine IC50 values. (B) In vitro PRC2 reactions with rPRC2-Ezh2 and 50 µM peptide substrate with increasing concentrations of EZHIP WT or M406E peptides (403-423). (C) Peptide pulldowns were performed as described in Figure 3E with EZHIP WT or M406E peptides (403-423). (D) Peptide pulldowns were performed as described in Figure 3E, with increasing concentrations of NaCl for washes. (E) Peptide pulldowns were performed as described in Figure 3E, except PRC2 was pre-incubated with 37.5 µM of either H3K27M 1-42 or 3x-FLAG peptide in the presence of 40 µM SAM before addition of biotinylated-EZHIP peptide and beads. (F) In vitro PRC2 assays using native PRC2 and oligonucleosome substrate as described in Figure 2B in the presence of increasing concentrations of full-length EZHIP or EZHIP peptide (403-423). (G) Immunoblots of whole cell extract (bottom) and eluates from FLAG M2 immunoprecipitation (top) from nuclear extracts of 293T cells expressing FLAG-tagged full length EZHIP or EZHIP fragments (1, 2, 4, 5, 8, 9, 10, 11) as shown in Figure 3H. Error bars in the figure represent standard deviation.

Supplementary Figure 4

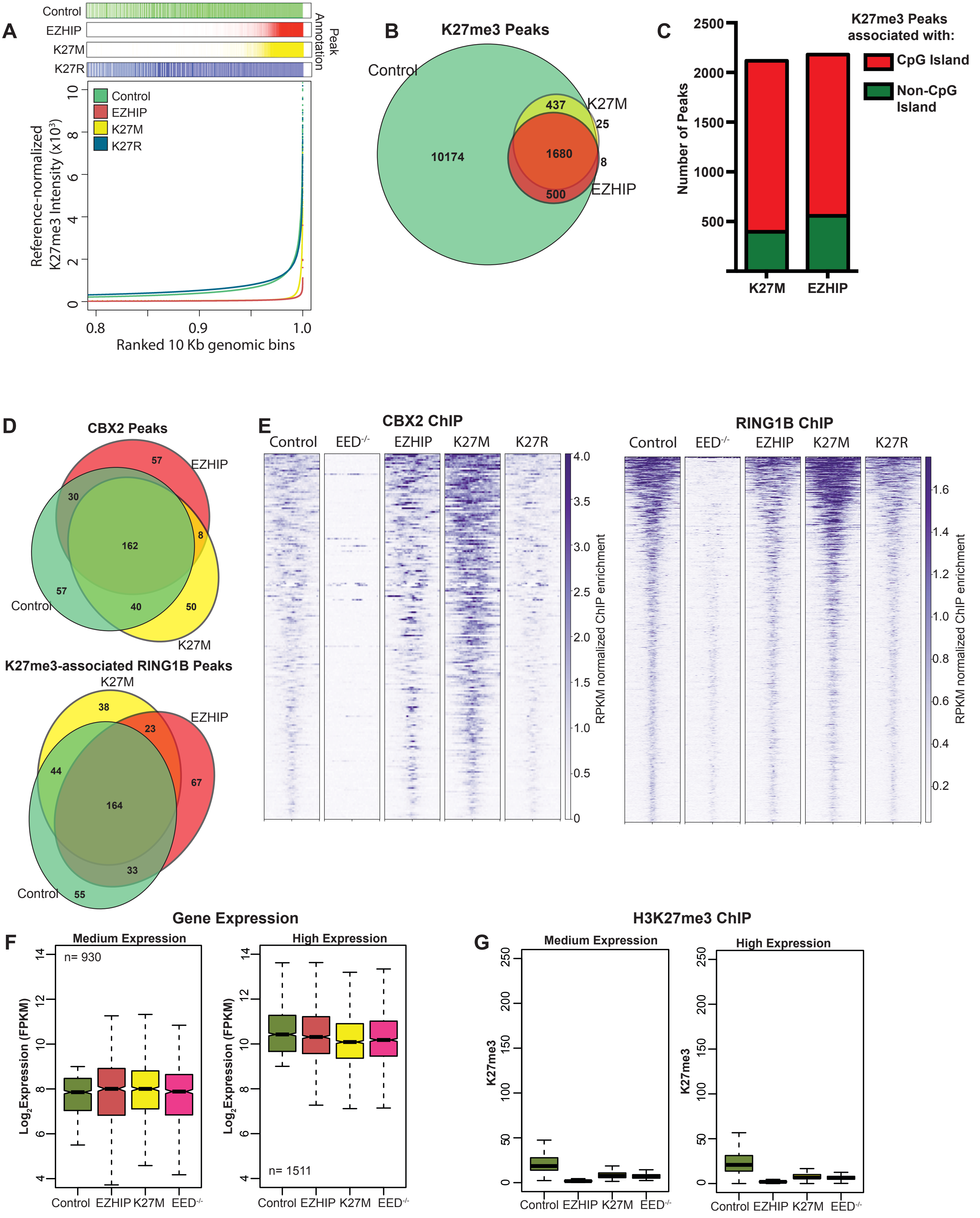

Supplementary Figure 4: "Residual" peaks of H3K27me3 are sufficient to recruit PRC1

(A) Distribution of reference-normalized H3K27me3 read density across the mouse genome. The genome was binned into 10 kb bins and ranked by their H3K27me3 densities (x-axis). Bins associated with H3K27me3 peaks are shown above the plot. (B) Venn diagram displaying the overlap between H3K27me3 peaks in MEFs expressing H3K27M, EZHIP and control cells. (C) Bar chart displaying the overlap of CpG islands and H3K27me3 peaks in cells expressing EZHIP or H3.3 K27M. (D)Venn diagram displaying the overlap between Cbx2 and Ring1b peaks. (E) Heatmap displaying the RPKM-normalized enrichment of Cbx2 and Ring1b at their corresponding peaks in control cells. (F) Boxplot displaying the expression of medium- (left) and high-expression (right) genes. (G) Boxplot displaying the Rx-normalized H3K27me3 ChIP enrichment at the promoters of genes in F. Center line in the boxplot represents the median, bottom and top of the box represents 25th and 75th quartiles; whiskers extend to 1.5 x interquartile range.

Supplementary Figure 5

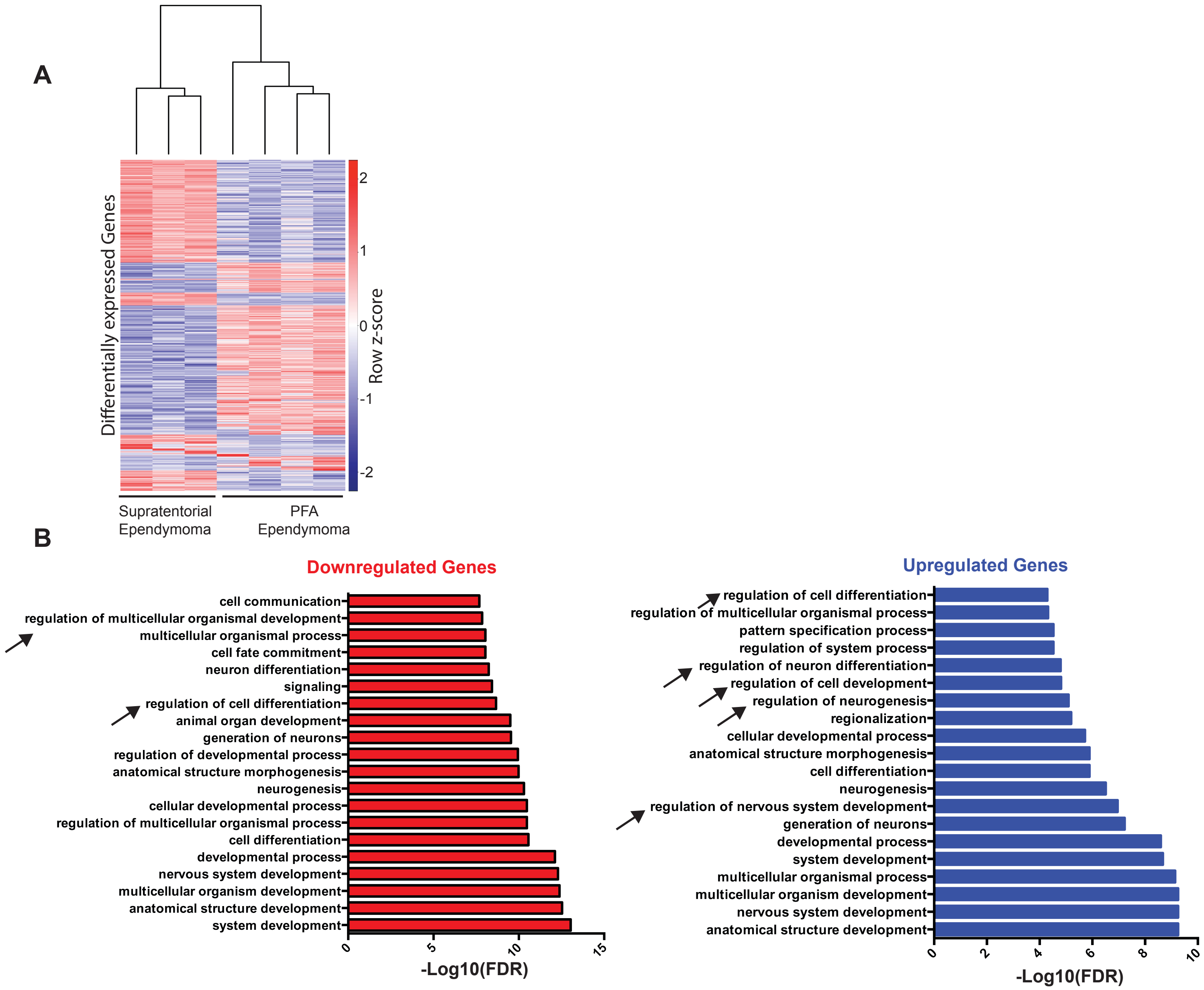

Supplementary Figure 5: Genes differentially expressed between PFA and Supratentorial ependymomas

(A) Heatmap displaying the expression profile of differentially expressed genes (>2x difference) in PFA and supratentorial ependymomas. We found 760 upregulated and 576 downregulated genes in PFA ependymomas relative to supratentorial tumors. (B) Top 20 GO terms enriched in differentially expressed genes between PFA and supratentorial ependymomas. Arrows indicate GO terms related to biological processes such as regulation of neurogenesis, cell differentiation and patterning
