# Supplementary material for: PFA ependymoma-associated protein EZHIP inhibits PRC2 activity through a H3 K27M-like mechanism
Source: Nat Commun. 2019 May 13;10:2146. doi: 10.1038/s41467-019-09981-6 (PMC6513997; doi:10.1038/s41467-019-09981-6)
Supplement: Supplementary file 3 — Description of Additional Supplementary Files [file 41467_2019_9981_MOESM3_ESM.pdf]

Description of Additional Supplementary Files

File name: Supplementary Data 1

Description: Histones extracted from 293T cells expressing H3.3WT, H3.3K27M, H3.3K27R, EZHIP WT, EZHIP R405E, H3.3-KLP Fusion proteins were subjected to bottom-up quantitative mass-spectrometry for measurement of post-translational modifications. Relative abundances of all histone modifications as calculated using EpiProfile based on chromatographic peak are summarized.

File name: Supplementary Data 2

Description: FLAG-tagged EZHIP was expressed in 293T cells and EZHIP-associated proteins were purified using FLAG affinity purification. Immunoprecipitated material was subjected to mass-spectrometry for protein identification and quantification. Identified proteins and their quantification are summarized in the dataset.
