# Supplementary material for: PFA ependymoma-associated protein EZHIP inhibits PRC2 activity through a H3 K27M-like mechanism
Source: Nat Commun. 2019 May 13;10:2146. doi: 10.1038/s41467-019-09981-6 (PMC6513997; doi:10.1038/s41467-019-09981-6)
Supplement: Supplementary file 6 — Reporting Summary [file 41467_2019_9981_MOESM6_ESM.pdf]

nature research

Corresponding author(s): Peter W. Lewis

Last updated by author(s): Mar 31, 2019

# Reporting Summary

Nature Research wishes to improve the reproducibility of the work that we publish. This form provides structure for consistency and transparency in reporting. For further information on Nature Research policies, see Authors & Referees and the Editorial Policy Checklist.

## Statistics

For all statistical analyses, confirm that the following items are present in the figure legend, table legend, main text, or Methods section.

| n/a | Confirmed | |
|---|---|---|
| ☐ | ☒ | The exact sample size (*n*) for each experimental group/condition, given as a discrete number and unit of measurement |
| ☒ | ☐ | A statement on whether measurements were taken from distinct samples or whether the same sample was measured repeatedly |
| ☐ | ☒ | The statistical test(s) used AND whether they are one- or two-sided<br>*Only common tests should be described solely by name; describe more complex techniques in the Methods section.* |
| ☒ | ☐ | A description of all covariates tested |
| ☒ | ☐ | A description of any assumptions or corrections, such as tests of normality and adjustment for multiple comparisons |
| ☐ | ☒ | A full description of the statistical parameters including central tendency (e.g. means) or other basic estimates (e.g. regression coefficient) AND variation (e.g. standard deviation) or associated estimates of uncertainty (e.g. confidence intervals) |
| ☐ | ☒ | For null hypothesis testing, the test statistic (e.g. *F*, *t*, *r*) with confidence intervals, effect sizes, degrees of freedom and *P* value noted<br>*Give P values as exact values whenever suitable.* |
| ☒ | ☐ | For Bayesian analysis, information on the choice of priors and Markov chain Monte Carlo settings |
| ☒ | ☐ | For hierarchical and complex designs, identification of the appropriate level for tests and full reporting of outcomes |
| ☒ | ☐ | Estimates of effect sizes (e.g. Cohen's *d*, Pearson's *r*), indicating how they were calculated |

*Our web collection on statistics for biologists contains articles on many of the points above.*

## Software and code

Policy information about availability of computer code

| | |
|---|---|
| Data collection | No software was used to collect data |
| Data analysis | For ChIP-Seq data: bowtie v1.0.0, IGV v2.4.16, mosaics-HMM v2.18, deeptools v3.0.2, R v3.5.0, bedtools v2.18, samtools v0.1.18<br>For RNA-Seq data: RSEM v1.2.4, EBSeq v1.20.0, R v3.5.0 |

For manuscripts utilizing custom algorithms or software that are central to the research but not yet described in published literature, software must be made available to editors/reviewers. We strongly encourage code deposition in a community repository (e.g. GitHub). See the Nature Research guidelines for submitting code & software for further information.

## Data

Policy information about availability of data

All manuscripts must include a data availability statement. This statement should provide the following information, where applicable:

- Accession codes, unique identifiers, or web links for publicly available datasets
- A list of figures that have associated raw data
- A description of any restrictions on data availability

The high throughput datasets generated in this study have been deposited to NCBI Gene Expression Omnibus (GSE124743, GSE124839 ; Figure 4). Publicly available data used in this study were downloaded from GEO (GSE87779, GSE89452, and GSE118954; Supplementary Figure 1, and Figure 5).

# Field-specific reporting

Please select the one below that is the best fit for your research. If you are not sure, read the appropriate sections before making your selection.

☒ Life sciences ☐ Behavioural & social sciences ☐ Ecological, evolutionary & environmental sciences

For a reference copy of the document with all sections, see nature.com/documents/nr-reporting-summary-flat.pdf

# Life sciences study design

All studies must disclose on these points even when the disclosure is negative.

| | |
|---|---|
| Sample size | No statistical method was used to determine sample size. Not applicable to the design of this study. |
| Data exclusions | None of the data was excluded from the study. |
| Replication | All in vitro reactions were repeated at least two times with similar results. Western blots were replicated multiple times (at least twice) with similar results. Representative images are shown in the figures. All ChIP-Seq experiments were performed once/twice. |
| Randomization | No randomization was performed. The conclusions made in this study were not affected by sample randomization. |
| Blinding | No blinding was performed. The results was measurements were not affected by the experimenters knowledge of sample identities. |

# Reporting for specific materials, systems and methods

We require information from authors about some types of materials, experimental systems and methods used in many studies. Here, indicate whether each material, system or method listed is relevant to your study. If you are not sure if a list item applies to your research, read the appropriate section before selecting a response.

## Materials & experimental systems

| n/a | Involved in the study | |
|---|---|---|
| ☐ | ☒ | Antibodies |
| ☐ | ☒ | Eukaryotic cell lines |
| ☒ | ☐ | Palaeontology |
| ☒ | ☐ | Animals and other organisms |
| ☒ | ☐ | Human research participants |
| ☒ | ☐ | Clinical data |

## Methods

| n/a | Involved in the study | |
|---|---|---|
| ☐ | ☒ | ChIP-seq |
| ☒ | ☐ | Flow cytometry |
| ☒ | ☐ | MRI-based neuroimaging |

## Antibodies

Antibodies used

H3K9me3: Active Motif 39161 (lot# 13509002)
H3K27ac: Active Motif 39133 (lot# 31814008)
H3K27me1: Millipore 07-448 (lot# 24439)
H3K27me2: Cell Signaling d18C8
H3K27me3: Cell Signaling C36B11 (lot# 8)
H3 general: Proteintech S2900-1
H4 general: Proteintech S2901-2
H3K36me3: Active motif 61101 (lot# 32412003)
H3K36me2: Cell Signaling 2901S (lot# 5)
FLAG: M2 Sigma Aldrich F1804
Kip75: Atlas hpa004003
EZH2: BD Biosciences 612666 (lot# 6230755)
EED: Active Motif clone 41D 61203
SUZ12: Cell Signaling D39F6 (lot# 3)
RBBP4: Proteintech 20364-I-ap (lot# 00022262)
RBBP4/6: LP bio AR-01-0178-200
Ring1b: Active motif 39663 (lot# 23012002)
Jarid2: Cell signaling 13594S (lot# 1)
Phf19: Cell signaling 77271S (lot# 1)
Brd4: Bethyl Laboratories a301-985a50 (lot# 6)
Cbx2: Bethyl Laboratories a302-524a (lot# 3)

Validation

Please see Materials and Methods. Additionally appropriate negative and positive controls were in ChIP-Seq experiments to validate the antibodies. Results of critical Immunoprecipitation and immunoblotting experiments that relied on antibodies were

further validated using Mass spectrometry (Supplementary Figure S1 and Figure 1).

# Eukaryotic cell lines

Policy information about cell lines

| | |
|---|---|
| Cell line source(s) | 293T cells from ATCC (CRL-3216). Mouse embryonic fibroblasts were isolated from EED flox/flox embryos at E13.4 stage. Cells were immortalized with SV-40 large T-antigen at passage-2, a single clone was selected for analyses. Low-passage (pass 5-20) cells were used for all analyses. |
| Authentication | The genetic background of mouse embryonic fibroblasts was validated by treating the EED flox/flox with lentiviruses for Cre-recominase that resulted in a loss of EED expression and H3K27 methylation (Figure 1, 4). |
| Mycoplasma contamination | All cell lines tested were negative for mycoplasma contamination using a PCR based method. |
| Commonly misidentified lines (See ICLAC register) | None of the commonly mis-identified cell lines was used in this study. |

# ChIP-seq

## Data deposition

☒ Confirm that both raw and final processed data have been deposited in a public database such as GEO.

☒ Confirm that you have deposited or provided access to graph files (e.g. BED files) for the called peaks.

Data access links
*May remain private before publication.*

https://www.ncbi.nlm.nih.gov/geo/query/acc.cgi?acc=GSE124743

Files in database submission

control_cbx2.bed
K27M_cbx2.bed
KIP75_cbx2.bed
KIP75_cbx2_mm9.bigwig
K27M_cbx2_mm9.bigwig
K27R_cbx2_mm9.bigwig
control_cbx2_mm9.bigwig
EEDnull_cbx2_mm9.bigwig
k27me3_enrichedRegions.bed
K27M_k27me3_peaks.bed
KIP75_k27me3_peaks.bed
KIP75_k27me3_mm9.bigwig
K27M_k27me3_mm9.bigwig
K27R_k27me3_mm9.bigwig
control_k27me3_mm9.bigwig
EEDnull_k27me3_mm9.bigwig
control_PRC1.bed
K27M_PRC1.bed
KIP75_PRC1.bed
KIP75_ring1b_mm9.bigwig
K27M_ring1b_mm9.bigwig
K27R_ring1b_mm9.bigwig
control_ring1b_mm9.bigwig
EEDnull_ring1b_mm9.bigwig
K27M_cbx2.bam
K27R_cbx2.bam
KIP75_cbx2.bam
control_cbx2.bam
EEDnull_cbx2.bam
K27M_k27me3.bam
K27R_k27me3.bam
KIP75_k27me3.bam
control_k27me3.bam
EEDnull_k27me3.bam
K27M_ring1b.bam
K27R_ring1b.bam
KIP75_ring1b.bam
control_ring1b.bam
EEDnull_ring1b.bam

Genome browser session
(e.g. UCSC)

not applicable. Data was visualized using IGV by loading bigwig files.

## Methodology

| | |
|---|---|
| Replicates | All ChIP-Seq experiments were performed 1 or 2 times. |
| Sequencing depth | ~25-40 million reads were mapped to reference genome for each samples. Libraries were sequenced with single-end, 50bp reads. |
| Antibodies | H3K27me3 Cell signaling 9733S (lot# 8)<br>Ring1b Active motif 39663 (lot# 23012002)<br>Cbx2 Bethyl Laboratories a302-524 (lot# 3) |
| Peak calling parameters | We used mosaics-HMM to identify peaks. See methods for details. |
| Data quality | Quality of data was ensured by using appropriate negative and positive controls. EED knockout cells were used as negative control for H3K27me3, CBX2 and RING1b ChIPs. Loss of signal enrichment was observed at previously known sites of PRC2 activity and PRC1 binding in EED-/- cells but not the control EED f/f cells (ex: HoxB locus). |
| Software | For ChIP-Seq data: bowtie v1.0.0, IGV v2.4.16, mosaics-HMM v2.18, deeptools v3.0.2, R v3.5.0, bedtools v2.18, samtools v0.1.18 . |
